# Influence of structure of iron nanoparticles in aggregates on their magnetic properties

**DOI:** 10.1186/1556-276X-6-527

**Published:** 2011-09-14

**Authors:** Dana Rosická, Jan Šembera

**Affiliations:** 1 Technical University of Liberec, Institute of Novel Technologies and Applied Informatics, Liberec, Czech Republic

**Keywords:** structure of aggregates, iron nanoparticles, aggregation, magnetic forces

## Abstract

Zero-valent iron nanoparticles rapidly aggregate. One of the reasons is magnetic forces among the nanoparticles. Magnetic field around particles is caused by composition of the particles. Their core is formed from zero-valent iron, and shell is a layer of magnetite. The magnetic forces contribute to attractive forces among the nanoparticles and that leads to increasing of aggregation of the nanoparticles. This effect is undesirable for decreasing of remediation properties of iron particles and limited transport possibilities. The aggregation of iron nanoparticles was established for consequent processes: Brownian motion, sedimentation, velocity gradient of fluid around particles and electrostatic forces. In our previous work, an introduction of influence of magnetic forces among particles on the aggregation was presented. These forces have significant impact on the rate of aggregation. In this article, a numerical computation of magnetic forces between an aggregate and a nanoparticle and between two aggregates is shown. It is done for random position of nanoparticles in an aggregate and random or arranged directions of magnetic polarizations and for structured aggregates with arranged vectors of polarizations. Statistical computation by Monte Carlo is done, and range of dominant area of magnetic forces around particles is assessed.

## 1 Introduction

Zero-valent iron nanoparticles (nZVI) composed of iron Fe^0 ^and its oxides are spherical particles with diameter approximately 40 nm and with a large specific surface. These particles are used for decontamination of groundwater and soil and especially for decontamination of organic pollutants such as halogenated hydrocarbons [1]. Nanoparticles migrate through the soil and can reach the contamination in situ. Properties of the nZVI and remediation possibilities depend on methods of production [2]. At the Technical University of Liberec, experiments with iron nanoparticles TODA, produced by the company Toda Kogyo Corp. [3], and with the nanoparticles NANOFER, produced by the company NANO IRON s.r.o. [4], are made. During a remedial intervention, transport of the iron nanoparticles is slowed down due to rapid aggregation of them. The rate of aggregation increases with growing concentration of particles in solution and with growing ionic strength of the solution [5]. With the word particle we mean both nanoparticles and aggregates. For preservation of the transport properties, it is advisable to stabilize the particles. A lot of methods of stabilization were published [6-10]. We simulate the transport of the iron nanoparticles and that is why we examine the interactions among them causing the aggregation. Models of aggregation of small particles were published in many articles (e.g., [11-13]). They are mostly based on the publications [14] and [15]. However, this generally used model is insufficient for our case. A surface charge established on the surface of particles causes repulsive electrostatic forces between them. The influence on the aggregation into the known aggregation model was implemented in submitted article [Rosicka and Sembera (2009)]. The iron particles corrode in the water, and this process causes change of the surface charge as well as the change of the rate of aggregation [16]. Because the particles are made from iron, they also have magnetic properties, which significantly affects the rate of aggregation [2,17-20]. In the previous work, the influence of magnetic forces on rate of aggregation of iron particles was assessed [21]. The magnetic forces have significant effect on aggregation. Size of this effect is dependent on the magnitude of polarization of nanoparticles, their directions and distances among nanoparticles or aggregates. We suppose same magnitude of vectors of polarization of all nanoparticles. Hence, there is presented evaluation of magnetic forces among particles depending only on the structure of aggregates, respectively, on directions of the vectors of polarization and distances between particles in this paper. In the future, the extended model of the aggregation of iron nanoparticles will be included into a solver of particle transport in groundwater. It would allow to simulate the transport of iron nanoparticles and to predict effciency of the remedial intervention. That could be useful for proposal of optimal remedial intervention which would enable to decontaminate an affected area effciently and economically.

## 2 Methods and models

### 2.1 Magnetic properties of nanoparticles

Because of the composition of nanoparticles, every nanoparticle has nonzero vector of magnetization. According to [19], the TODA iron nanoparticles with diameter 40 nm have saturation magnetization 570 kA/m. This is value for composition of the nanoparticles 14.3% of Fe_0 _and 85.7% of Fe_3_O_4_. We use these data for our model. Therefore, we suppose the same magnitude of vector of polarization for all nanoparticles. Our model of magnetic field around the iron nanoparticle is based on the model of magnetic field around a magnet described in [22]. The electromagnetic potential in the point **r **near a permanent magnet of volume *V *is equal to

(1) $$\phi \left(\text{r}\right)=\underset{V}{\int }\frac{MR}{R^{3}}\text{d}V,$$

where **M **is the vector of magnetic polarization at the point d*V *, the vector **R **is the difference between the source of magnetic field d*V *and the point **r**, *R *is the length of **R**.

Intensity of the magnetic field **H **can be subsequently computed as

(2) H(r)=-grad(ϕ(r)).

Finally, the magnetic force between the source of the intensity of magnetic field **H **and a permanent magnet of volume  Ṽ with the vector of polarization **M**_0 _at the point **r **is equal to

(3) $$F\left(r\right)=-\int_{Ṽ}\left(M_{0}\cdot \;\text{grad}\right)H\left(r\right)\text{d}V.$$

In prior work [21], we derived scalar potential of the magnetic field around one homogeneous spherical iron nanoparticle with radius *a *located at the point [0, 0, 0]:

(4) ϕ(r)=M∫ 02π∫ 0π∫ 0a(x3-r′cos(θ))r′2 sin(θ)(x12+x22+x32-r2)23dr′dθdφ,

where *a *is the radius of the nanoparticle, and [*x*_1_, *x*_2_, *x*_3_] are the coordinates of the point **r**. Here, the direction of the vector of polarization **M **is set to the direction *x*_3_, *M *is the magnitude of the vector **M**.

From Equations 2 and 3, the analytical computation of magnetic force between two iron nanoparticles can be obtained. Since the nanoparticles aggregate, the magnetic force between aggregates is required to derive. One aggregate can be composed of millions of nanoparticles. It would be time-consuming and very difficult to analytically compute these forces. As a consequence, the forces are computed numerically, either as a sum of magnetic forces between every nanoparticle in one aggregate with every nanoparticle in second aggregate or as one magnetic force between two averaged aggregates. Averaged aggregate is a big homogeneous particle with direction of polarization **M***_A _*computed as a vector sum of vectors of polarization of all nanoparticles in the aggregate *M_A _*computed as an average of magnitudes of all nanoparticles divided by number of nanoparticles in the aggregate *n*:

(5) $$M_{A}=\frac{\sum_{i=1}^{n}M_{i}}{n}.$$

### 2.2 Unstructured model of aggregates

**Figure 1:**
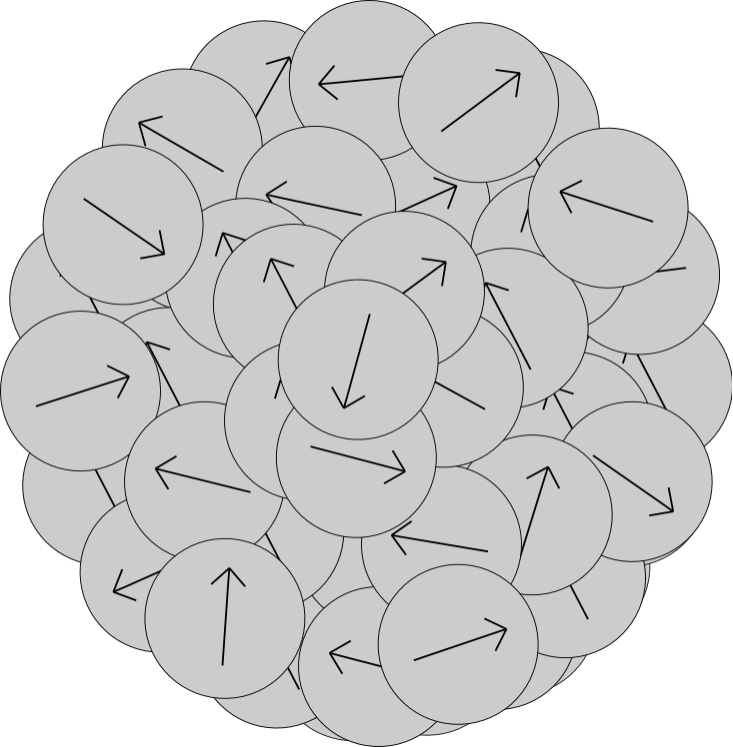
**Unstructured model of aggregate with random directions of vectors of polarization of nanoparticles creating the aggregate**.

**Figure 2:**
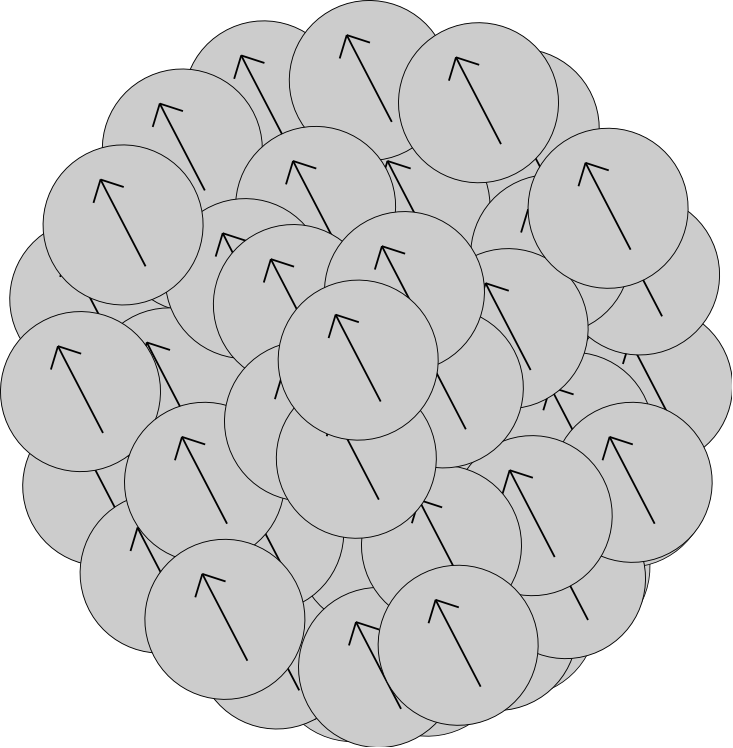
**Unstructured model of aggregate with the same direction of polarization of nanoparticles creating the aggregate**.

The rate of aggregation depends on the vectors of polarization of aggregating particles and on distance between the particles. Rate of aggregation changes with changing structure of ordering of nanoparticles in the aggregates. In this section, we imagine the model of structure of aggregate as sphere with randomly located nanoparticles in the aggregate either with random directions of polarization of every nanoparticle (Figure 1) or with the same direction of polarization of all nanoparticles in the aggregate (Figure 2).

### 2.3 Structured model of aggregates

**Figure 3:**
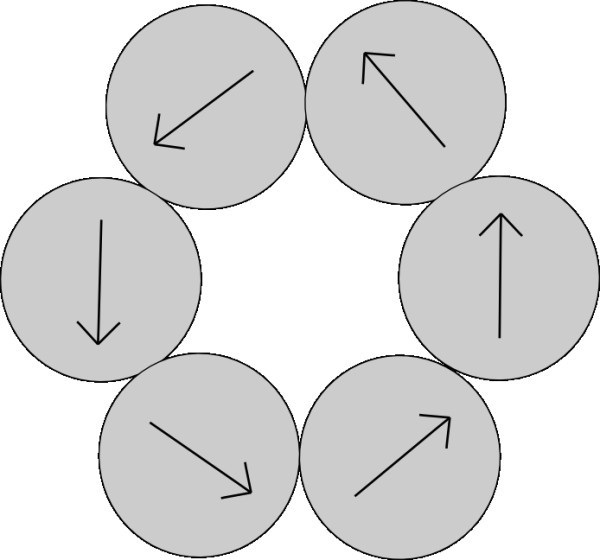
**Structured model of aggregate in shape of circle with directions of polarization respecting the shape**.

**Figure 4:**
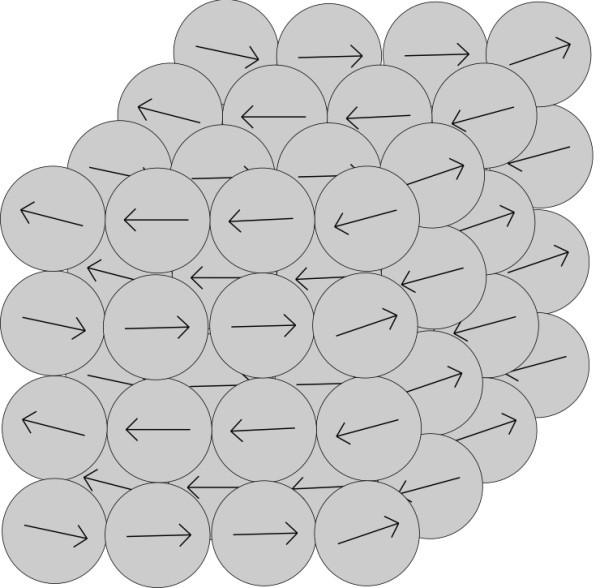
**Structured model of aggregate in shape of cube with directions of polarization respecting the shape**.

**Figure 5:**
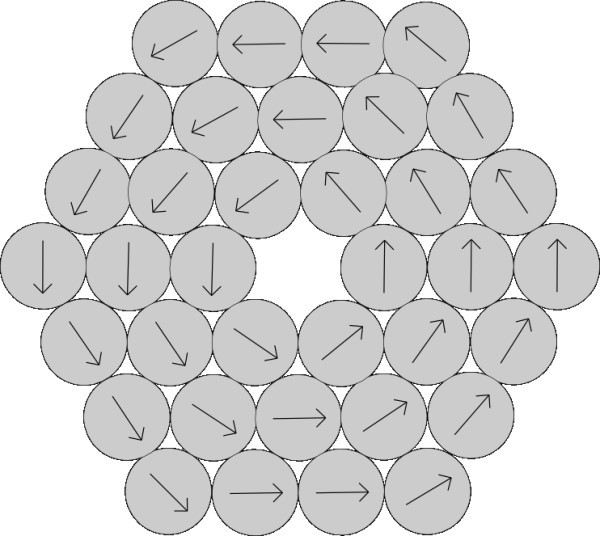
**Structured model of aggregate in shape of hexagon with directions of polarization respecting the shape**.

In this section, we suppose structured ordering of nanoparticles in the aggregate. Vectors of polarization of the nanoparticles respect the structure. According to our observations of behavior of spherical magnets, the magnets create clusters with least energy. With small number of magnets, the most created clusters are circles formed by chains. We considered also other structured models of aggregates - cubic and honeycomb. Directions of vectors of polarization of nanoparticles in these models of structured aggregates are shown in Figures 3, 4 and 5. On the basis of results in Section 3.2, it was redundant to create more structured forms.

**Figure 6:**
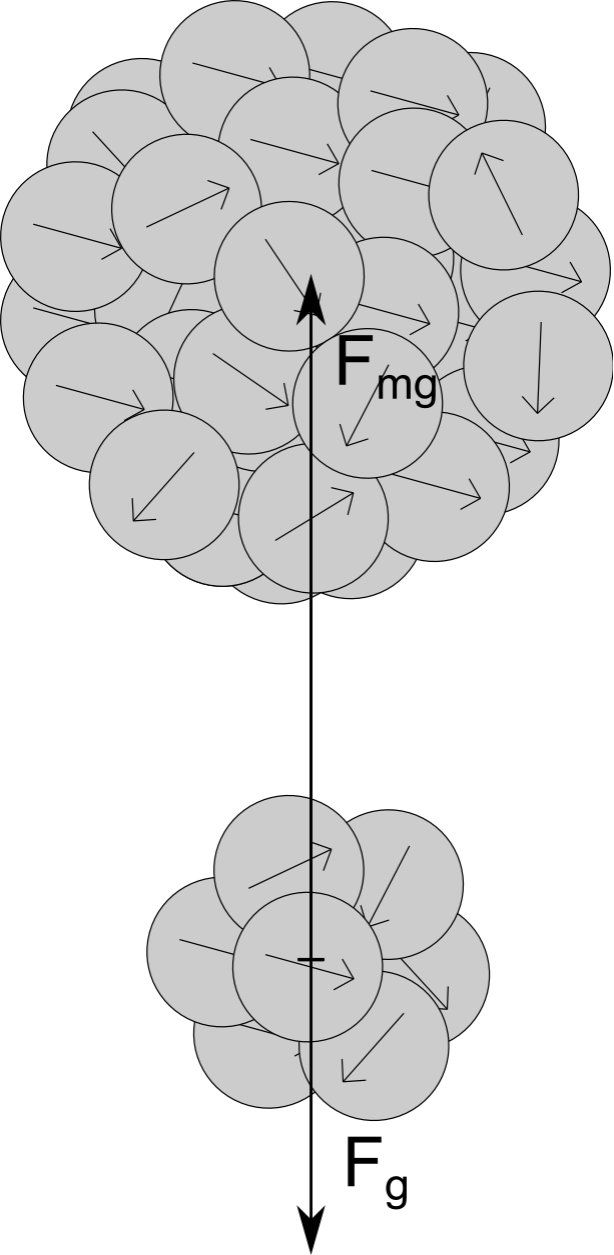
**Comparing of attractive magnetic force and counteracting gravitation force**.

In the prior work [21], we used a term "effective range". It is range in which the magnetic field around one aggregate attract an aggregate placed in the range. In this article, we plot the influence of magnetic forces as one number - limit distance. This dimension also expresses the range of the magnetic forces among particles. Up to this distance from the center of an aggregate, the attractive magnetic forces cause the aggregation of the aggregate and a particle placed in the range. So in range larger than the limit distance, other forces outweight the magnetic forces. For evaluation of this parameter, we compared the magnetic forces with a gravitation force affected on the particle, according to scheme Figure 6.

## 3 Results and discussion

### 3.1 Unstructured model of aggregates

#### 3.1.1 Random directions of polarization of nanoparticles in aggregates

**Figure 7:**
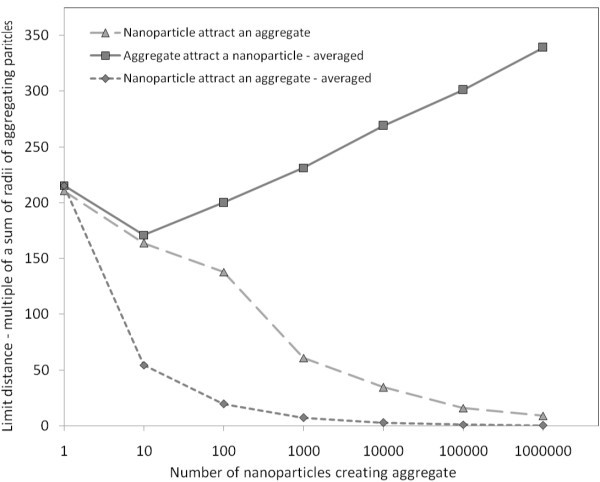
**Graph of limit distances of interacting aggregate with one nanoparticle**. Solid line represents attractive magnetic force between an aggregate and a nanoparticle on which gravitation force effects. The dashed lines represent attractive magnetic force between a nanoparticle attracting an aggregate. Square marks represent data computed by averaging, triangle marks stand for sum of magnetic forces of a nanoparticle and all nanoparticles in an aggregate.

Unstructured model of ordering of nanoparticles in an aggregate has been computed by averaging and by summation of magnetic forces between every nanoparticle in first aggregate and every nanoparticle in second aggregate. In Figure 7, the comparison of averaging and summation is plotted in 2D for interactions between one nanoparticle and an aggregate. Comparison can be done by comparing of limit distances or effective range of magnetic field around the particle.

**Figure 8:**
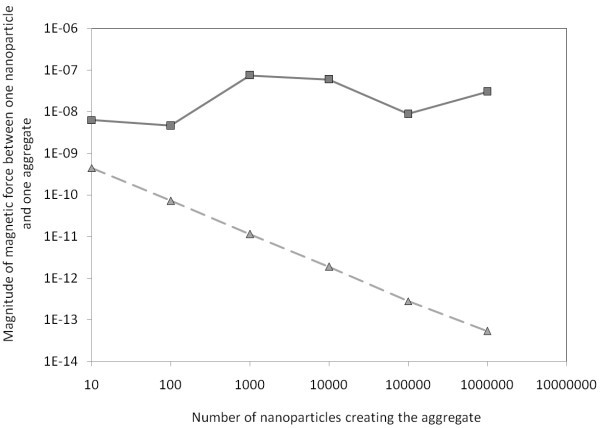
**Graph of magnitude of magnetic force between one nanoparticle and an aggregate**. Particles are attached to each other what means that the distance between particles is equal to the sum of radii of both particles. The triangle marks represent data computed by averaging of the positions, directions, and magnitudes of vectors of polarization of all nanoparticles in the examined aggregate. The magnetic force is then computed between the nanoparticle and the averaged aggregate. The squared marks represent data computed by summation of all interactions between the examined nanoparticle and every nanoparticle from the examined aggregate. Every nanoparticle has random position and direction of vector of polarization. The particles are attached to each other, otherwise distance between the particles is the sum of radii of particles.

**Figure 9:**
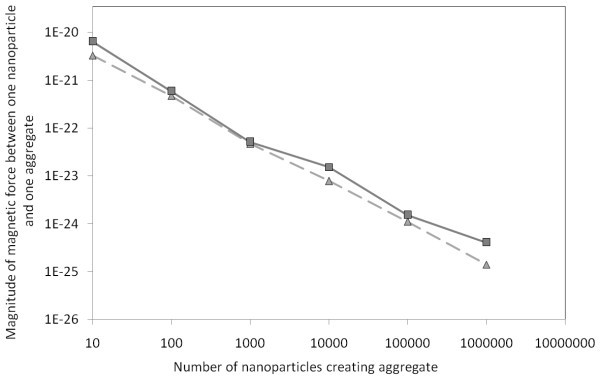
**Graph of magnitude of magnetic force between one nanoparticle and an aggregate**. Particles are in distance to each other of one thousand multiple of the radius of examined aggregate. The triangle marks represent data computed by averaging of the positions, directions, and magnitudes of vectors of polarization of all nanoparticles in the examined aggregate. The magnetic force is then computed between the nanoparticle and the averaged aggregate. The squared marks represent data computed by summation of all interactions between the examined nanoparticle and every nanoparticle from the examined aggregate. Every nanoparticle has random position and direction of vector of polarization.

The limit distance estimated by averaging decreases more rapidly with growing aggregate than properly computed magnetic forces. It means that for large aggregates the averaging cannot be done, for the case of attached particles. There is a lot of nanoparticles in the big aggregate, and nanoparticles closer to the other particle have larger influence on the magnetic force between the aggregate and the examining nanoparticle. Comparison of averaged magnetic forces and summed magnetic forces for attached particles is done in Table 1. The plotted data can be seen in the Figure 8. However, in long distance between the particle and an aggregate, the difference between averaging and summation is insignificant. The distances between nanoparticles in examined aggregate are negligible in comparison with distance between the aggregate and examined nanoparticle, as you can see in the Table 2 and in the Figure 9. These data are computed for distance between particles of 1000 multiply of radius of the aggregate.

**Table 1:** Table of magnitudes of magnetic forces between interacting nanoparticle and aggregate

*i*	Averaged *F*_mg_	Deviation	Summed *F*_mg_	Deviation
1	1.4·10^-9^	2.5·10^-9^	6.3·10^-9^	1.1·10^-9^
10	4.4·10^-10^	1.9·10^-10^	6.3·10^-9^	8.4·10^-9^
100	7.2·10^-11^	2.7·10^-11^	4.6·10^-9^	5.4·10^-9^
1,000	1.1·10^-11^	3.0·10^-12^	7.4·10^-8^	1.1·10^-7^
10,000	1.9·10^-12^	6.0·10^-13^	5.9·10^-8^	7.0·10^-8^
1,000,000	2.8·10^-13^	1.1·10^-13^	8.7·10^-9^	1.0·10^-8^
1,000,000	5.4·10^-14^	2.2·10^-14^	3.0·10^-8^	4.9·10^-8^

Symbol *i *stands for number of nanoparticles in the aggregate. Averaged *F*_mg _is the magnetic force between the nanoparticle and an aggregate with averaged vectors of polarizations and destinations of nanoparticles in the aggregate. Summed *F*_mg _is magnetic force between the nanoparticle and the aggregate computed by summation of magnetic forces between the nanoparticle and all the nanoparticles in the aggregate. Symbol Deviation stands for averaged value of absolute deviations of data points from their mean value

**Table 2:** Table of magnitudes of magnetic forces between interacting nanoparticle and aggregate

*i*	Averaged *F*_mg_	Deviation	Summed *F*_mg_	Deviation
1	2.0·10^-20^	1.0·10^-20^	3.1·10^-20^	5.5·10^-21^
10	3.3·10^-21^	2.3·10^-21^	6.5·10^-21^	4.0·10^-21^
100	4.7·10^-22^	2.8·10^-22^	5.9·10^-22^	2.2·10^-22^
1,000	4.7·10^-23^	1.7·10^-23^	5.1·10^-23^	3.0·10^-23^
10,000	7.9·10^-24^	4.0·10^-24^	1.5·10^-23^	8.5·10^-24^
1,000,000	1.1·10^-24^	5.6·10^-25^	1.5·10^-24^	1.1·10^-24^
1,000,000	1.4·10^-25^	7.1·10^-26^	4.1·10^-25^	2.0·10^-25^

Symbol *i *stands for number of nanoparticles in the aggregate. Averaged *F*_mg _is the magnetic force between the nanoparticle and an aggregate with averaged vectors of polarizations and destinations of nanoparticles in the aggregate. Summed *F*_mg _is magnetic force between the nanoparticle and the aggregate computed by summation of magnetic forces between the nanoparticle and all the nanoparticles in the aggregate. Symbol Deviation stands for averaged value of absolute deviations of data points from their mean value. Distance between the nanoparticle and the aggregate is one thousand multiply of radius of the aggregate

**Figure 10:**
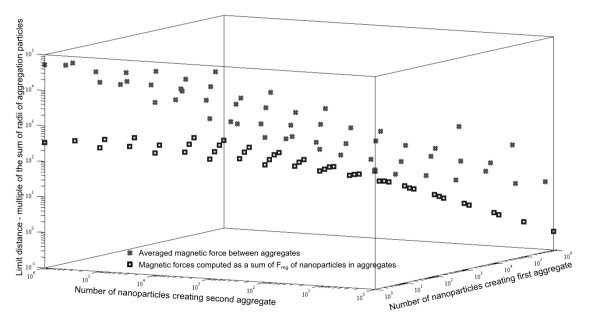
**Graph of limit distances of two interacting aggregates**. Crosses represent limit distances computed by averaging of magnetic forces. Squares stand for sum of magnetic forces of every nanoparticle from one aggregate and every nanoparticle in second aggregate. On the left side of the graph, gravitation force effects on the smaller aggregate, on the right side, gravitation force effects on the bigger aggregate.

You can also see the results for magnetic forces between two interacting aggregates (Figure 10). The computations are done by the averaging and the summation again. The conclusion is the same as for the case of interacting aggregate with nanoparticle. In the case of long distances among particles, computing by averaging can be used. In the case of short distances, the time-consuming computation by summation has to be done.

On the basis of known concentration of particles, we are able to estimate the approximate distances among particles and to choose the right model of computation of magnetic forces and limit distances among the particles.

#### 3.1.2 The same directions of polarization of all nanoparticles in aggregates

**Figure 11:**
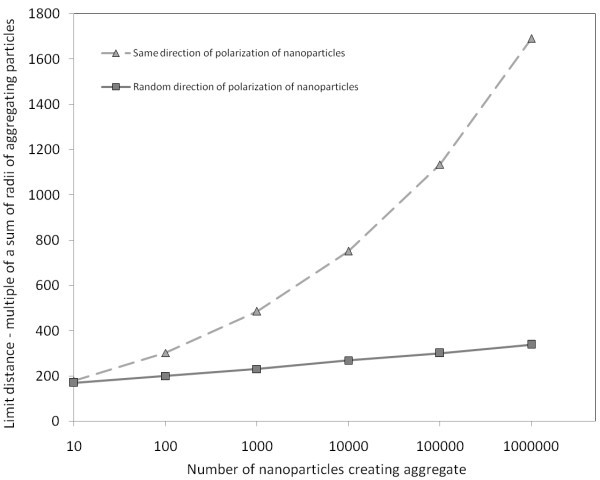
**Graph of limit distances of interacting aggregate and nanoparticle representing comparison of the same and random polarization of nanoparticles in the aggregate**. Square marks represent the data of interaction between the nanoparticle and the aggregate with random directions of polarization of nanoparticles in the aggregate. Triangle marks represent the data of interaction between the nanoparticle and the aggregate with the same direction of polarization of nanoparticles in the aggregate. Both the random and the same structured aggregates interactions were computed by summation.

We examined structure of aggregates in which vectors of polarization of all nanoparticles were the same in magnitude and direction, distribution of nanoparticles in the aggregate was unstructured. Comparison of limit distances for unstructured model with random directions of polarization of nanoparticles in the aggregate and for structured model with the same directions of polarization is shown in Figure 11.

### 3.2 Structured model of aggregates

Further we examined model of structure of aggregates in which nanoparticles creating the aggregate are distributed to a structure and vectors of polarization respect the structure. According to our observations of behavior of spherical magnets, the magnets create clusters with less energy in which vectors of polarization subtract one another and final vector of polarization approaches to zero. Consequently, in the case of structured ordering of nanoparticles, the magnetic forces among the particles have not any influence on the aggregation of particles. In the following table, magnitude of magnetic force between a nanoparticle and a cubic aggregate attached to each other is presented. Directions of polarization of nanoparticles in the aggregate are set according to the Figure 4. Results show that the magnetic force between structured particles approaches to zero. Results of cubic structure are visible in Table 3.

**Table 3:** Table of magnetic forces between one nanoparticle and cubic aggregate

*i*	*|F*_mg_|	*|F_g_|*
1	2.4·10^-38^	2.0·10^-18^
8	4.1·10^-38^	1.6·10^-17^
125	6.6·10^-40^	2.5·10^-16^
1,000	7.7·10^-41^	2.0·10^-15^
10,648	8.4·10^-42^	2.2·10^-14^
97,336	5.7·10^-42^	2.0·10^-13^
1,000,000	5.0·10^-42^	2.0·10^-12^

The particles are attached to each other. Symbol *i *stands for number of nanoparticles in the cubic aggregate, *|F*_mg_*| *is magnitude of magnetic force between nanoparticle and the aggregate, and *|F_g_| *stands for magnitude of gravitation force between nanoparticle and the aggregate

Even though the distance between the particles is the smallest possible, the magnetic forces between the particles are negligible in comparison with gravitation forces. For structured aggregates with polarization of nanoparticles respecting the structure, the magnetic forces have insignificant influence on the rate of aggregation of the particles.

## 4 Conclusion

We examined the influence of structure of nanoparticles in aggregates on the resulting magnetic field around the aggregates. The comparing parameter was size of magnetic forces among particles and the limit distance. According to these two connected parameters, we are able to estimate the degree of influence of the magnetic forces on rate of aggregation of particles. We analyzed magnetic properties of unstructured aggregates with randomly distributed nanoparticles with random direction of polarization in the aggregates and of structured model of aggregates with orderly distributed nanoparticles and with arranged directions of polarization. In the case of structured model, resulting magnetic forces approach to zero. Since we suppose that magnetic field of iron particles has significant influence on the rate of aggregation [19], the structured model is not sufficient. Hence, we assume damaged structure of aggregates. In our future work, the unstructured model will be compared with experimental data of aggregation of iron particles.

## Competing interests

The authors declare that they have no competing interests.

## Authors' contributions

DR carried out the study of influence of structure of nanoparticles in aggregates and drafted the manuscript. JŠ contributed to conception of the study and to interpretation of data, and revised the manuscript. Both authors read and approved the final manuscript.
